# An Exploration of Methods to Resolve Inconsistent Self-Reporting of Chronic Conditions and Impact on Multimorbidity in the Canadian Longitudinal Study on Aging

**DOI:** 10.1177/08982643231215476

**Published:** 2023-11-28

**Authors:** Alessandra T. Andreacchi, Alberto Brini, Edwin Van den Heuvel, Graciela Muniz-Terrera, Alexandra Mayhew, Philip St John, Lucy E. Stirland, Lauren E. Griffith

**Affiliations:** 1 Department of Health Research Methods, Evidence, and Impact, 62703 McMaster University, Hamilton, ON, Canada; 2 Department of Mathematics and Computer Science, 3169 Eindhoven University of Technology, Eindhoven, The Netherlands; 3 Heritage College of Osteopathic Medicine, 43973 Ohio University, Athens, OH, USA; 4 Labarge Centre for Mobility in Aging, 3710 McMaster University, Hamilton, ON, Canada; 5 McMaster Institute for Research on Aging, 3710 McMaster University, Hamilton, ON, Canada; 6 Section of Geriatric Medicine, Max Rady College of Medicine, 8664 University of Manitoba, Winnipeg, MB, Canada; 7 Centre for Clinical Brain Sciences, University of Edinburgh, Edinburgh, Scotland, UK; 8 Global Brain Health Institute, 8785 University of California, San Francisco, CA, USA

**Keywords:** Canadian longitudinal study on aging, morbidity, chronic disease, CLSA

## Abstract

**Objectives:**

To quantify inconsistent self-reporting of chronic conditions between the baseline (2011–2015) and first follow-up surveys (2015–2018) in the Canadian Longitudinal Study on Aging (CLSA), and to explore methods to resolve inconsistent responses and impact on multimorbidity.

**Methods:**

Community-dwelling adults aged 45–85 years in the baseline and first follow-up surveys were included (*n* = 45,184). At each survey, participants self-reported whether they ever had a physician diagnosis of 35 chronic conditions. Identifiable inconsistent responses were enumerated.

**Results:**

32–40% of participants had at least one inconsistent response across all conditions. Illness-related information (e.g., taking medication) resolved most inconsistent responses (>93%) while computer-assisted software asking participants to confirm their inconsistent disease status resolved ≤53%. Using these adjudication methods, multimorbidity prevalence at follow-up increased by ≤1.6% compared to the prevalence without resolving inconsistent responses.

**Discussion:**

Inconsistent self-reporting of chronic conditions is common but may not substantially affect multimorbidity prevalence. Future research should validate methods to resolve inconsistencies.

## Background

Multimorbidity—commonly defined by the presence of 2 or more chronic conditions (Boyd & Fortin, 2010)—is an established risk factor for reduced quality of life (Fortin et al., 2007), functional disability (Griffith et al., 2017; St. John et al., 2019), and premature mortality (Gijsen et al., 2001). Multimorbidity is strongly related to age and mid- to older-aged adults living in high-income countries have approximately 3 chronic conditions on average (Ofori-Asenso et al., 2019; St. John et al., 2021). Most research on multimorbidity thus far has been cross-sectional (Prados-Torres et al., 2014), and the nature of multimorbidity progression over time is unclear (Vetrano et al., 2018). Large population-based longitudinal studies are becoming increasingly important for multimorbidity research, particularly for research on aging (National Institute on Aging, 2007; Raina et al., 2019); they can monitor trends in chronic conditions and multimorbidity over time as well as capture trajectories and changes in the profiles of aging (Martin & Romero Ortuño, 2019).

Epidemiological research studies often utilize surveys that allow participants to self-report chronic conditions because they are relatively easy to implement and inexpensive (Fortin et al., 2017). A largely overlooked concern with using self-report in longitudinal studies is the consistency with which participants report their chronic condition status in baseline surveys and subsequent follow-up survey waves (Quiñones et al., 2019). Health surveys often ask participants whether they have *ever* been diagnosed by a physician with a particular chronic condition, and by definition, chronic conditions are incurable and persist throughout an individual’s life despite treatment to manage symptoms. Thus, the general consensus within the research community has been to view an initial self-reported diagnosis as the ‘truth’ and carry the initial response forward to the subsequent survey wave (Fisher et al., 2005). In actuality, participants’ responses may change when surveyed in subsequent waves. Improving the consistency of self-reported chronic conditions may improve the precision of prevalence and incidence estimates, including estimates of multimorbidity. This improvement, in turn, may result in more accurate estimates of the health care costs attributed to multimorbidity and improve the accuracy of studies exploring the associations of multimorbidity.

There may be several reasons for inconsistent reporting of chronic conditions over time. First, participants in longitudinal studies may not be aware of a chronic condition they have been diagnosed with, or they may believe that they do not have the condition. Second, the symptom severity may vary over time, and the individual may be more likely to report a condition which is symptomatic at that time, and less likely to report it when the symptom burden is lower. Third, a participant may believe the condition is cured and is no longer present or were misdiagnosed. Finally, diagnostic criteria may change over time, resulting in differential reporting. Inconsistent self-reporting of chronic conditions in longitudinal studies is not well investigated (Quiñones et al., 2020). Few international studies have explored this issue and suggest inconsistent self-reporting of any chronic condition occurs in up to 22%–43% of participants, highlighting a substantial methodological concern (Beckett et al., 2000; Cigolle et al., 2016; Jensen et al., 2019; Klabunde et al., 2005; Quiñones et al., 2019; Ryu et al., 2018). The extent of inconsistent self-reporting may also vary across chronic conditions and socio-demographic groups including by age, socioeconomic position, and level of cognitive impairment (Beckett et al., 2000; Cigolle et al., 2016; Jensen et al., 2019; Klabunde et al., 2005; Quiñones et al., 2019; Ryu et al., 2018). Only one known study has attempted to devise a method to adjudicate inconsistent responses by using disease-related information including medication use, treatment for the disease, or time of diagnosis to verify an individual’s disease status (Cigolle et al., 2016). A better understanding of various approaches to resolving inconsistent responses is an essential methodological consideration to inform the design of surveys for longitudinal studies. Furthermore, no known studies have addressed how inconsistencies in the self-reporting of chronic conditions may impact multimorbidity prevalence.

Our study has three aims. First, we aim to quantify the inconsistent self-reporting of chronic conditions between the baseline (2011–2015) and first follow-up survey (2015–2018) in the Canadian Longitudinal Study on Aging (CLSA). Second, we aim to understand the socio-demographic, health-related, and cognitive factors that are associated with inconsistent self-reporting of chronic conditions. Third, we also aim to explore methods to resolve inconsistencies in self-reported chronic conditions through additionally collected information that can inform disease status, and to investigate the impact of each adjudication method on the prevalence of multimorbidity.

## Methods

### Study Design and Population

The CLSA is a large nationally generalizable, longitudinal research platform that includes 51,338 community-dwelling adults from the 10 Canadian provinces. The complete methods and eligibility criteria have been described previously (Raina et al., 2019). Tracking cohort participants (*n* = 21,241) were randomly selected from the 10 provinces and completed interviews by telephone. Comprehensive cohort participants (*n* = 30,097) were randomly selected from within 25–50 km of 11 data collection sites in seven Canadian provinces (Raina et al., 2019). Participants in the Comprehensive cohort completed in-person interviews in their homes, as well as in-depth physical assessments and biological specimen collection at one of the data collection sites (Raina et al., 2019). Participants were recruited from 2010 to 2015, the baseline survey was conducted from 2011 to 2015, and the first follow-up survey was conducted from 2015 to 2018 (Raina et al., 2019). Comprehensive and Tracking cohort participants who completed the baseline and first follow-up surveys were included but analyzed separately resulting in a final sample size of 27,765 participants in the Comprehensive cohort and 17,419 participants in the Tracking cohort.

### Measurement of Chronic Conditions

At baseline and follow-up interviews, participants were asked to self-report their disease status for various chronic conditions. Chronic conditions were defined in CLSA questionnaires as “long-term conditions” which are expected to last or have lasted 6 months or more and have been diagnosed by a health professional (Canadian Longitudinal Study on Aging (CLSA), 2015, 2018). Participants were asked the same question at each interview, *“has a doctor ever told you that you have ___ disease?”* and response options were *“yes,” “no,*” *“don’t know/no answer,”* or *“refused.”* We focus this study on the chronic conditions most commonly reported in multimorbidity indices and consulted two clinicians with expertise in geriatrics and multimorbidity research (P.S. and L.S.) to determine the most relevant conditions to aging which persist throughout an individual’s life despite treatment to manage symptoms (Diederichs et al., 2011; Johnston et al., 2019). The following 35 chronic conditions were considered: (1) osteoarthritis in the hand; (2) osteoarthritis in the hip; (3) osteoarthritis in the knee; (4) rheumatoid arthritis; (5) osteoporosis; (6) back problems; (7) asthma; (8) chronic obstructive pulmonary disorder (including emphysema, chronic bronchitis, or chronic lung changes due to smoking); (9) angina; (10) myocardial infarction; (11) heart disease (including congestive heart failure); (12) hypertension; (13) peripheral vascular disease; (14) hypothyroidism (under-active); (15) hyperthyroidism (over-active); (16) diabetes; (17) stroke or cardiovascular accident; (18) transient ischemic attack; (19) Parkinsonism disease; (20) multiple sclerosis; (21) epilepsy; (22) migraine headaches; (23) intestinal or stomach ulcer; (24) bowel disorder; (25) bowel incontinence; (26) urinary incontinence; (27) cataracts; (28) glaucoma; (29) macular degeneration; (30) mood disorder (including depression, bipolar disorder, mania, dysthymia); (31) clinical depression; (32) anxiety; (33) dementia including Alzheimer’s disease; (34) kidney disease; (35) cancer (excluding non-melanoma skin cancer).

### Types of Inconsistent Self-Reported Responses

Longitudinal patterns of chronic disease for a given participant may be clinically consistent (i.e., always affirmative, always negative, or negative then affirmative) or clinically inconsistent (i.e., affirmative then negative). For each of the 35 chronic conditions, two types of clinically inconsistent self-reported responses between baseline and follow-up survey were considered in this study, in line with previous studies (Beckett et al., 2000; Cigolle et al., 2016; Jensen et al., 2019; Klabunde et al., 2005; Quiñones et al., 2019; Ryu et al., 2018). The first type of inconsistent self-reported response occurred when a participant’s response to having a particular chronic condition was affirmative at baseline then negative at follow-up (responded *“yes”* then *“no”*). The second type of inconsistent self-reported response occurred when a participant’s response to having a particular chronic condition was affirmative at baseline but unknown at follow-up (responded *“yes”* then *“don’t know/no answer”* or *“refused”*) (see Supplemental Materials (e)Figure 1 for a visual schematic). These types of inconsistent responses represent those that are identifiable; other types of inconsistent responses are possible (e.g., incorrectly reporting negative at baseline and affirmative at follow-up) but are not possible to identify in this study.

### Socio-Demographic and Health-Related Variables

Selected a priori based on previous literature (Beckett et al., 2000; Cigolle et al., 2016; Jensen et al., 2019; Klabunde et al., 2005; Quiñones et al., 2019; Ryu et al., 2018), the following variables were collected at baseline and considered to understand the socio-demographic and health-related factors associated with inconsistent self-reporting of chronic conditions: age (45–54; 55–64; 65–74; 75+) sex (male; female), number of chronic conditions, race (White; not White), immigrant status (non-immigrant; immigrant), province (Alberta; British Columbia; Manitoba; New Brunswick [Tracking cohort only]; Newfoundland and Labrador; Nova Scotia; Ontario; Prince Edward Island [Tracking cohort only]; Quebec; Saskatchewan [Tracking cohort only]), education (less than secondary school; secondary school graduation; some post-secondary education; post-secondary degree/diploma), household income (less than $20,000; $20,000 or more, but less than $50,000; $50,000 or more, but less than $100,000; $100,000 or more, but less than $150,000; $150,000 or more), marital status (single/never married; married/common-law; widowed; divorced/separated), interview language (English; French), visit to a general practitioner in the past 12 months (yes; no), self-reported general health (fair/poor; good; very good; excellent), and cognitive impairment scores. Cognitive impairment tests included the first and second recall of the RAVLT (Rey Auditory Verbal Learning Test) T-score (Rey, 1964), Animal Naming T-score (Goodglass & Kaplan, 1972), and the Mental Alternation Test (MAT) T-score (Teng, 1995) with each test score categorized as moderate to severe impairment, moderate impairment, mild impairment, below average or borderline impairment, and average.

### Adjudication Methods to Resolve Inconsistent Self-Reported Responses

Three separate methods were used to adjudicate or ‘resolve’ inconsistent self-reported responses across all 35 chronic conditions among participants in the Comprehensive cohort only, as additional information required to resolve inconsistent responses was not collected in the Tracking cohort. These methods were employed to determine the impact of inconsistent responses on the prevalence of multimorbidity (defined as >1, >2 and >3 chronic conditions).

In Method A, participants who inconsistently responded affirmative at baseline then negative at follow-up were probed by computer-assisted survey software to verify their disease status through an additional question at follow-up. These participants were asked *“in your baseline (your first) CLSA interview, you indicated YES to the question that you had been told by a doctor that you had ___ disease. Since that interview, has the diagnosis changed?”* and response options were *“yes”*, *“no,*” *“don’t know/no answer,”* or *“refused.”* Only participants who inconsistently responded affirmative at baseline and then negative at follow-up for a particular condition were asked this verification question. Therefore, participants who responded affirmative at baseline and then unknown at follow-up could not be resolved using Method A. Participants who answered *“no”* to this verification question, confirming their inconsistent self-reported disease status, had their response at follow-up adjudicated and changed to *“yes.”* Participants who answered *“yes”* or *“don’t know/no answer/refused”* to this question kept their responses at follow-up unchanged as *“no”* despite being inconsistent.

In Method B*,* illness-related information collected at baseline was used to verify the responses of participants who inconsistently responded affirmative at baseline and then negative or unknown at follow-up for a given condition. Illness-related information was only collected if a participant indicated having a chronic condition. If illness-related information collected at baseline could support the diagnosis, then participants had their response at follow-up adjudicated and changed to *“yes.”* Similar to the method used to resolve inconsistent responses in a previous study (Cigolle et al., 2016), illness-related information considered for each chronic condition included (i) age of diagnosis, (ii) whether the participant currently or had ever taken medication to treat the condition, or (iii) whether the participant currently or had ever undergone non-pharmacological treatment to treat the condition (Canadian Longitudinal Study on Aging (CLSA), 2015, 2018). A visual schematic depicting Method A and Method B to resolve inconsistent responses is available in Supplemental Materials (e)Figures 2 and 3, respectively, and additional information used to adjudicate an inconsistent response for each condition is available in eTable 1.

In Method C, a participant’s baseline response was considered to be their “true” disease status and was carried forward as their response at follow-up. It was assumed that no inconsistent responses existed using this method.

### Statistical Analysis

For each chronic condition, frequencies and proportions of both types of inconsistent self-reported responses (i.e., affirmative then negative, and affirmative then unknown) were calculated in the Comprehensive and Tracking cohorts, separately. The total number of each type of inconsistent response across all 35 chronic conditions was then enumerated for each participant in the Comprehensive and Tracking cohorts. To examine factors associated with inconsistent self-reporting of chronic conditions, socio-demographic and health-related factors were compared across participants with no inconsistent responses and participants with at least one inconsistent response across all chronic conditions. Inconsistent responses that were affirmative at baseline and then negative at follow-up were the focus of this and all subsequent analyses as this type of inconsistency was more common and represents a definitive misreporting of the condition. Factors were compared between participants with no and at least one inconsistent response using counts and percentages for categorical factors, and medians and interquartile ranges (IQR) otherwise. To assess whether differences in factors existed across those with no and at least one inconsistent response, p-values from Pearson’s chi-square tests and ANOVA, and standardized differences (<.1 signifying no difference) (Austin, 2009) were used accordingly. Multivariable logistic regression analyses were conducted to estimate odds ratios (OR) and 95% confidence intervals (CI) for the association between factors and having at least one inconsistent response across all chronic conditions (reference: no inconsistent responses). Multivariable logistic regression models were considered; one was unadjusted, and another was adjusted for age and sex.

The proportion of inconsistent responses that could be resolved using Method A and Method B, separately, was estimated in the Comprehensive cohort for each chronic condition. Lastly, the prevalence of multimorbidity in the Comprehensive cohort before resolving inconsistent responses was compared to the prevalence after applying Methods A, B, and C to resolve inconsistent responses. The prevalence of multimorbidity was determined using common definitions including >1, >2, and >3 of the 35 chronic conditions (Fortin et al., 2012; Johnston et al., 2019). CLSA analytical weights were used in all descriptive and regression analyses to reflect the eligible Canadian population in the geographic areas around the data collection sites. This study was approved by the Hamilton Integrated Research Ethics Board (Ethics certificate #: 7424). Participants of the CLSA provided written informed consent to participate.

## Results

### Enumerating Inconsistent Self-Reported Chronic Conditions

Inconsistent self-reported responses that were affirmative at baseline and then negative at follow-up were more common than inconsistent responses that were affirmative and then unknown across all conditions (Table 1). In the Comprehensive cohort, the five conditions with the greatest proportion of affirmative then negative inconsistent responses were back problems (4.2%), clinical depression (3.5%), cataracts (3.0%), hypertension (2.2%), and osteoarthritis in the hand (2.2%). In the Tracking cohort, the top five conditions were back problems (7.3%), osteoarthritis in the hand (4.4%), migraine headaches (3.8%), and osteoarthritis in the knee (3.0%) and hip (2.9%) (Note: clinical depression was not collected in the Tracking cohort).

**Table 1.** Frequency and Proportion of Participants With Inconsistent Self-reported Chronic Conditions in the Canadian Longitudinal Study on Aging Comprehensive (*n* = 27,765) and Tracking Cohorts (*n* = 17,429).

	Comprehensive cohort				Tracking cohort			
	Affirmative at baseline, negative at follow-up^a^		Affirmative at baseline, unknown at follow-up^b^		Affirmative at baseline, negative at follow-up^a^		Affirmative at baseline, unknown at follow-up^b^	
Chronic conditions	N	%	N	%	N	%	N	%
Angina	189	0.7	25	0.1	211	1.2	10	0.1
Anxiety	393	1.4	26	0.1	361	2.1	4	0.0
Asthma	297	1.1	45	0.2	322	1.8	5	0.0
Back problems	1174	4.2	65	0.2	1272	7.3	26	0.1
Bowel disorder	457	1.6	35	0.1	324	1.9	11	0.1
Bowel incontinence	188	0.7	1	0.0	178	1.0	0	0.0
Cancer (excluding non-melanoma)	221	0.8	15	0.1	223	1.3	22	0.1
Cataracts	839	3.0	39	0.1	445	2.6	16	0.1
Chronic obstructive pulmonary disorder	310	1.1	32	0.1	286	1.6	9	0.1
Clinical depression	958	3.5	112	0.4	N/C	N/C	N/C	N/C
Diabetes	416	1.5	20	0.1	260	1.5	8	0.0
Dementia including Alzheimer’s disease	19	0.1	2	0.0	12	0.1	1	0.0
Epilepsy	34	0.1	0	0.0	14	0.1	1	0.0
Glaucoma	342	1.2	21	0.1	137	0.8	9	0.1
Heart disease	472	1.7	32	0.1	423	2.4	20	0.1
Hypertension	622	2.2	60	0.2	453	2.6	13	0.1
Hyperthyroidism	128	0.5	26	0.1	107	0.6	19	0.1
Hypothyroidism	176	0.6	61	0.2	165	0.9	54	0.3
Intestinal or stomach ulcer	419	1.5	26	0.1	384	2.2	5	0.0
Kidney disease	182	0.7	8	0.0	127	0.7	3	0.0
Macular degeneration	181	0.7	30	0.1	139	0.8	9	0.1
Migraine headaches	458	1.6	20	0.1	661	3.8	6	0.0
Mood disorder (depression, bipolar, mania, dysthymia)	562	2.0	30	0.1	506	2.9	13	0.1
Myocardial infarction	86	0.3	28	0.1	101	0.6	13	0.1
Multiple sclerosis	19	0.1	1	0.0	12	0.1	2	0.0
Osteoarthritis in hand	602	2.2	111	0.4	769	4.4	40	0.2
Osteoarthritis in hip	425	1.5	103	0.4	512	2.9	27	0.2
Osteoarthritis in knee	481	1.7	132	0.5	530	3.0	41	0.2
Osteoporosis	401	1.4	68	0.2	429	2.5	34	0.2
Parkinsonism/disease	5	0.0	0	0.0	3	0.0	0	0.0
Peripheral vascular disease	367	1.3	20	0.1	407	2.3	19	0.1
Rheumatoid arthritis	154	0.6	39	0.1	315	1.8	14	0.1
Stroke	87	0.3	12	0.0	51	0.3	2	0.0
Transient ischemic attack	125	0.5	34	0.1	127	0.7	11	0.1
Urinary incontinence	588	2.1	4	0.0	460	2.6	3	0.0

*Notes:* N/C = not collected.

^a^Depicts a participant who self-reported “yes” to having a given chronic condition at baseline and then “no” at follow-up.

^b^Depicts a participant who self-reported “yes” to having a given chronic condition at baseline and then “don’t know/no answer,” or “refused” at follow-up.

Approximately 32% of participants in the Comprehensive cohort had at least one affirmative at baseline then negative at follow-up inconsistent response across all conditions; 21.3% of participants had one, 6.4% had two, and 1.8% had three of these inconsistent responses (Table 2). The proportion of participants with at least one affirmative at baseline then negative at follow-up inconsistent response was slightly higher in the Tracking cohort (40%) with 25.5%, 9.6%, and 3.2% having one, two, and, three of these inconsistent response, respectively. The proportion of participants with at least one affirmative at baseline then unknown at follow-up inconsistent response was 4.0% and 2.4% in the Comprehensive and Tracking cohorts, respectively.

**Table 2.** Frequency and Proportion of the Total Number of Inconsistent Responses per Participant Across all 35 Chronic Conditions in the Canadian Longitudinal Study on Aging Comprehensive (*n* = 27,765) and Tracking Cohorts (*n* = 17,429).

	Comprehensive cohort				Tracking cohort			
Total number of inconsistent responses per participant	Affirmative at baseline, negative at follow-up^a^		Affirmative at baseline, unknown at follow-up^b^		Affirmative at baseline, negative at follow-up^a^		Affirmative at baseline, unknown at follow-up^b^	
	N	%	N	%	N	%	N	%
0	18,858	67.9	26,663	96.0	10,343	60.0	16,831	97.6
1	6423	23.1	957	3.4	4403	25.5	381	2.2
2	1789	6.4	113	0.4	1650	9.6	25	0.1
3	503	1.8	28	0.1	546	3.2	10	0.1
4	132	0.5	4	0.0	203	1.2	1	0.0
5	34	0.1	0	0.0	65	0.4	1	0.0
6	17	0.1	0	0.0	28	0.2	0	0.0
7	6	0.0	0	0.0	9	0.1	0	0.0
8	2	0.0	0	0.0	1	0.0	0	0.0
9	1	0.0	0	0.0	1	0.0	0	0.0
10	0	0.0	0	0.0	0	0.0	0	0.0
**Total**	27,765	100.0	27,765	100.0	17,249	100.0	17,249	100.0

^a^Depicts a participant who self-reported “yes” to having a given chronic condition at baseline and then “no” at follow-up.

^b^Depicts a participant who self-reported “yes” to having a given chronic condition at baseline and then “don’t know/no answer,” or “refused” at follow-up.

### Socio-Demographic and Health-Related Factors Associated With Inconsistent Self-Reported Chronic Conditions

Table 3 describes baseline socio-demographic and health-related factors across participants who had no and at least one affirmative at baseline and then negative at follow-up inconsistent responses in the Comprehensive cohort (Supplemental Materials eTable 2 for the Tracking cohort). The median number (IQR) of chronic conditions among participants with no and at least one affirmative at baseline and then negative at follow-up inconsistent responses was 2 (1–4) and 4 (3–6), respectively, in the Comprehensive cohort and 2 (1–4) and 4 (2–6), respectively, in the Tracking cohort. There were no differences in cognitive impairment scores between participants who had no and at least one affirmative at baseline and negative at follow-up inconsistent responses as evidenced by standardized differences in test scores <.1 (Supplemental Materials eTables 3–4) (Austin, 2009). Table 4 presents OR and 95% CI for the association between each factor and at least one affirmative at baseline and then negative at follow-up inconsistent response across all chronic conditions (reference: no inconsistent response) in the Comprehensive cohort (Supplemental Materials eTable 5 for the Tracking cohort, eTable 6 for cognitive test scores). In age-adjusted models, females compared to males had greater odds of reporting at least one affirmative at baseline and then negative at follow-up inconsistent response compared to no inconsistent responses (OR = 1.28, 95% CI: 1.21–1.36). In sex-adjusted models, participants of older age categories compared to ages 45–54 had greater odds of reporting at least one affirmative and then negative inconsistent response (ages 55–64: OR = 1.47 95% CI: 1.36–1.59; ages 65–74: OR = 2.02 95% CI: 1.87–2.19; ages 75+: OR = 2.57 95% CI: 2.36–2.81). In models adjusted for sex and age, participants who had less education (compared to post-secondary degree/diploma), lower household income (compared to $150,000 or more), were widowed (compared to married/common law), visited their general practitioner in the past 12 months (compared to no visit), and had self-reported lower general health (compared to self-reporting “excellent”) had greater odds of reporting at least one affirmative at baseline and then negative at follow-up inconsistent response. A single increase in the number of self-reported chronic conditions was associated with 1.45 (95% CI: 1.43–1.48) times greater odds of reporting at least one affirmative at baseline and then negative at follow-up inconsistent response.

**Table 3.** Baseline Socio-demographic and Health-related Factors of Participants in the Canadian Longitudinal Study on Aging Comprehensive Cohort (*n* = 27,765) who had No and At Least One Affirmative at Baseline and Negative at Follow-up Inconsistent Response Across all 35 Chronic Conditions.

Socio-demographic and health-related factors	No inconsistent responses *N* = 18,858 (67.9%)		≥1 inconsistent response *N* = 8907 (32.1%)		Chi-square p-value	Standardized difference	Overall *N* = 27,765	
	N	%	N	%			N	%
Sex								
Male	9624	51.0	4008	45.0	<.0001	.12	13,632	49.1
Female	9234	49.0	4899	55.0		.12	14,133	50.9
Missing	0	0.0	0	0.0		.00	0	0.0
Age group								
45–54	5523	29.3	1622	18.2	<.0001	.26	7145	25.7
55–64	6468	34.3	2808	31.5		.06	9276	33.4
65–74	4265	22.6	2516	28.2		.13	6781	24.4
75+	2602	13.8	1961	22.0		.22	4563	16.4
Missing	0	0.0	0	0.0		.00	0	0.0
Race								
White	18,042	95.7	8561	96.1	.009	.02	26,603	95.8
Not white	799	4.2	335	3.8		.02	1134	4.1
Missing	17	0.1	11	0.1		.01	28	0.1
Immigrant status								
Non-immigrant	15,502	82.2	7275	81.7	0.6	.01	22,777	82.0
Immigrant	3354	17.8	1631	18.3		.01	4985	18.0
Missing	2	0.0	1	0.0		.00	3	0.0
Province								
Alberta	1878	10.0	892	10.0	<.0001	.00	2770	10.0
British Columbia	3977	21.1	1807	20.3		.02	5784	20.8
Manitoba	1752	9.3	1123	12.6		.11	2875	10.4
Newfoundland and Labrador	1434	7.6	559	6.3		.05	1993	7.2
Nova Scotia	1944	10.3	833	9.4		.03	2777	10.0
Ontario	4013	21.3	1982	22.3		.02	5995	21.6
Quebec	3860	20.5	1711	19.2		.03	5571	20.1
Missing	0	0.0	0	0.0		.00	0	0.0
Highest level of education								
Less than secondary school	806	4.3	574	6.4	<.0001	.10	1380	5.0
Secondary school graduation	1691	9.0	848	9.5		.02	2539	9.1
Some post-secondary education	1269	6.7	753	8.5		.07	2022	7.3
Post-secondary degree/diploma	15,067	79.9	6714	75.4		.11	21,781	78.4
Missing	25	0.1	18	0.2		.02	43	0.2
Household income								
Less than $20,000	770	4.1	533	6.0	<.0001	.09	1303	4.7
$20,000 or more, but less than $50,000	3495	18.5	2122	23.8		.13	5617	20.2
$50,000 or more, but less than $100,000	6204	32.9	3082	34.6		.04	9286	33.4
$100,000 or more, but less than $150,000	3789	20.1	1429	16.0		.11	5218	18.8
$150,000 or more	3493	18.5	1124	12.6		.16	4617	16.6
Missing	1107	5.9	617	6.9		.04	1724	6.2
Marital status								
Single/Never married	1658	8.8	739	8.3	<.0001	.02	2397	8.6
Married/Common law	13,444	71.3	5864	65.8		.12	19,308	69.5
Widowed	1459	7.7	999	11.2		.12	2458	8.9
Divorced/Separated	2292	12.2	1302	14.6		.07	3594	12.9
Missing	5	0.0	3	0.0		.00	8	0.0
Interview language								
English	15,142	80.3	7275	81.7	.006	.04	22,417	80.7
French	3716	19.7	1632	18.3		.04	5348	19.3
Missing	0	0.0	0	0.0		.00	0	0.0
Contact with general practitioner in past 12 months								
No	2090	11.1	609	6.8	<.0001	.15	2699	9.7
Yes	16,472	87.3	8179	91.8		.15	24,651	88.8
Missing	296	1.6	119	1.3		.02	415	1.5
Self-reported general health								
Fair/Poor	1295	6.9	1043	11.7	<.0001	.14	2338	8.4
Good	5147	27.3	2897	32.5		.11	8044	29.0
Very good	8133	43.1	3526	39.6		.07	11,659	42.0
Excellent	4273	22.7	1432	16.1		.17	5705	20.5
Missing	10	0.1	9	0.1		.02	19	0.1

**Table 4.** Weighted Odds Ratios and 95% Confidence Intervals for the Association Between Socio-demographic and Health-related Factors, and Odds of at Least One Affirmative at Baseline and Then Negative at Follow-up Inconsistent Response (Reference: No Inconsistent Responses) Across all 35 Chronic Conditions Among Participants in the Canadian Longitudinal Study on Aging Comprehensive Cohort (*n* = 27,765).

Socio-demographic and health-related factors	Sample size	Unadjusted			Adjusted for age and sex (continuous)		
		OR	Lower CI	Upper CI	OR	Lower CI	Upper CI
Sex	27,765						
Male		Ref			Ref		
Female		1.30	1.23	1.38	1.28	1.21	1.36
Age group	27,765						
45–54		Ref			Ref		
55–64		1.47	1.36	1.58	1.47	1.36	1.59
65–74		2.04	1.89	2.21	2.02	1.87	2.19
75+		2.60	2.38	2.83	2.57	2.36	2.81
Race	27,737						
White		Ref			Ref		
Not white		.86	.74	1.00	.96	.83	1.12
Immigrant status	27,762						
Non-immigrant		Ref			Ref		
Immigrant		1.01	.94	1.09	.96	.89	1.04
Province	27,765						
Alberta		.87	.77	.98	.94	.84	1.06
British Columbia		.91	.83	.99	.91	.83	.99
Manitoba		1.29	1.17	1.43	1.30	1.17	1.44
Newfoundland and Labrador		.81	.72	.92	.82	.72	.93
Nova Scotia		.92	.82	1.02	.93	.83	1.03
Ontario		Ref			Ref		
Quebec		.93	.85	1.01	.92	.84	1.00
Education	27,722						
Less than secondary school graduation		1.80	1.59	2.04	1.36	1.19	1.55
Secondary school graduation, no post-secondary education		1.20	1.08	1.32	1.07	.97	1.18
Some post-secondary education		1.35	1.21	1.50	1.25	1.12	1.40
Post-secondary degree/diploma		Ref			Ref		
Household income	26,041						
Less than $20,000		2.38	2.06	2.76	1.80	1.54	2.09
$20,000 or more, but less than $50,000		1.93	1.75	2.12	1.36	1.23	1.51
$50,000 or more, but less than $100,000		1.55	1.42	1.69	1.26	1.15	1.38
$100,000 or more, but less than $150,000		1.16	1.05	1.29	1.08	.98	1.20
$150,000 or more		Ref			Ref		
Marital status	27,757						
Single/Never married		1.06	.95	1.17	1.11	.99	1.23
Married/Common law		Ref			Ref		
Widowed		1.70	1.55	1.87	1.01	.91	1.12
Divorced/Separated		1.35	1.24	1.46	1.23	1.13	1.34
Interview language	27,765						
French		.95	.89	1.03	.94	.87	1.01
English		Ref			Ref		
Visit to general practitioner in past 12 months	27,350						
No		Ref			Ref		
Yes		1.74	1.56	1.94	1.48	1.33	1.65
Self-reported general health	27,746						
Fair/Poor		2.53	2.25	2.83	2.58	2.29	2.90
Good		1.70	1.56	1.85	1.70	1.56	1.86
Very good		1.30	1.20	1.41	1.31	1.20	1.42
Excellent		Ref			Ref		
Number of chronic conditions	27,765	1.45	1.43	1.47	1.45	1.43	1.48

*Notes:* OR = odds ratio; CI = confidence interval; ref = reference.

### Resolving Inconsistent Self-Reported Chronic Conditions

The proportion of each type of inconsistent response (affirmative at baseline and then negative at follow, and affirmative and then unknown) that were resolved using Methods A and B are presented in Supplemental Materials eTable 7. Most (>93%) inconsistent responses were resolved using Method B for all chronic conditions (except for osteoporosis where 13% of affirmative at baseline and then negative at follow-up inconsistent responses and 0% of affirmative at baseline and then unknown at follow-up inconsistent responses were resolved). Fewer affirmative at baseline and then negative at follow-up inconsistent responses were resolved using Method A (<53%) compared to Method B. The proportion resolved using Method A varied depending on the chronic condition, ranging from 0% and 7% resolved for Parkinsonism and myocardial infarction, respectively, to 53% for hypothyroidism.

### Impact of Resolving Inconsistent Self-Reported Chronic Conditions on the Prevalence of Multimorbidity

Table 5 demonstrates the prevalence of multimorbidity at follow-up (using definitions of >1, >2, and >3 chronic conditions) before any inconsistent responses were resolved and after Methods A, B, and C were applied to resolve inconsistencies. Compared to the multimorbidity prevalence (>1 chronic conditions) before resolving inconsistent responses, the prevalence increased by 0.6% and 1.6% on the absolute scale when using Methods A and B, respectively, to adjudicate inconsistent responses. When the participant's baseline disease status was carried forward to follow-up and inconsistencies were ignored (Method C), the prevalence of multimorbidity increased by 3.8%.

**Table 5.** Prevalence of Multimorbidity at Follow-up (2015–2018) Before and After Resolving Inconsistent Self-reporting of Chronic Conditions Using Methods A, B and C in the Canadian Longitudinal Study on Aging Comprehensive Cohort (*n* = 27,765).

	Prevalence before resolving inconsistent responses		Prevalence after method A^a^		Prevalence after method B^b^		Prevalence after method C^c^		Difference before and after method A		Difference before and after method B		Difference before and after method C	
	N	%	N	%	N	%	N	%	Absolute (%)	Relative (%)	Absolute (%)	Relative (%)	Absolute (%)	Relative (%)
>1 chronic condition	20,856	75.1	21,029	75.7	21,311	76.8	21,921	79.0	0.6	0.8	1.6	2.2	3.8	5.1
>2 chronic conditions	16,719	60.2	16,959	61.1	17,389	62.6	18,229	65.7	0.9	1.4	2.4	4.0	5.4	9.0
>3 chronic conditions	12,827	46.2	13,089	47.1	13,552	48.8	14,528	52.3	0.9	2.0	2.6	5.7	6.1	13.3

^a^In Method A, participants who responded affirmative at baseline and then negative at follow-up were probed by computer-assisted survey software to answer an additional question to resolve their inconsistent response.

^b^In Method B, participants who inconsistently responded affirmative at baseline and then negative or unknown at follow-up had illness-related information collected at baseline consulted to resolve their inconsistent response.

^c^In Method C, participants who inconsistently responded affirmative at baseline and then negative or unknown at follow-up had their baseline response carried forward as their response at follow-up.

## Discussion

Inconsistencies in the longitudinal self-reporting of chronic conditions were common among a nationally representative sample of Canadian adults aged 45–85 years at baseline. Approximately 32% of participants in the Comprehensive cohort reported affirmative and then negative to at least 1 of 35 chronic conditions between the CLSA baseline and follow-up survey approximately 3 years later. An adjudication method that directly asked participants with inconsistent responses to confirm their disease status resolved up to 53% of inconsistent responses across chronic conditions. A less conservative method using illness-related information collected at baseline, such as age at diagnosis and medication use, resolved most (>93%) inconsistent responses. Adjudicating inconsistent responses using either method did not substantially alter the prevalence of multimorbidity at follow-up compared to the prevalence before resolving inconsistencies (0.6–1.6%), although previous studies have noted differences in the prevalence for individual chronic conditions may be more substantial (i.e., up to 14% for stroke) (Cigolle et al., 2016). Carrying forward a participant’s initial disease status at baseline to subsequent interview waves as is typically done in epidemiological studies resulted in the largest difference in prevalence of 3.8%.

### Comparison of Findings to Previous Literature

The percentage of participants inconsistently reporting any chronic condition was 32% and 40% in the CLSA Comprehensive and Tracking Cohorts, respectively, which is in line with previous studies whose estimates ranged from 22% to 43% (Cigolle et al., 2016; Quiñones et al., 2019; Ryu et al., 2018). Prior studies differ with respect to the demographic profile of their study populations and the types of chronic conditions considered, making it difficult to infer the types of chronic conditions most inconsistently reported in longitudinal studies on aging. For example, an American study by Cigolle and colleagues using 1995–2010 waves of the Health and Retirement study with adults aged 51 years and older (*n* = 24,156) found arthritis and hypertension were the two most inconsistently reported conditions out of the seven evaluated; (Cigolle et al., 2016) these were among the top five of 35 conditions most inconsistently reported in our study. Conversely, a study by Jensen and colleagues using data from 2013 and 2017 waves of the Danish Health and Morbidity Surveys on participants aged 16+ (*n* = 2297) found hypertension was one of the least inconsistently reported conditions out of the 18 evaluated. (Jensen et al., 2019) Having a mental disorder <6 months was also among the most inconsistently reported conditions in this Danish study, which was similar to our findings of clinical depression being among the top five inconsistently reported chronic conditions in the Comprehensive cohort. A better understanding of the types of conditions most likely to be inconsistently reported and potential causes in longitudinal studies of older adult populations can inform the design of survey questionnaires.

Socio-demographic and health-related factors associated with longitudinal inconsistent self-reporting of chronic conditions in our study were comparable to those in previous studies (Cigolle et al., 2016; Jensen et al., 2019; Klabunde et al., 2005; Quiñones et al., 2019; Ryu et al., 2018), although most presented results adjusted for different socio-demographic and health-related variables, preventing direct comparisons. No known study evaluated the association between the number of chronic conditions and inconsistent reporting. The number of chronic conditions was strongly associated with inconsistent reporting. In exploratory analyses (data not shown), most associations between socio-demographic and health-related factors and the odds of inconsistent reporting were attenuated or became null when additionally adjusting for the number of chronic conditions, which may be hypothesized as a mediator of these associations. These exploratory analyses suggest a greater number of chronic conditions may be driving the association of socio-demographic and health-related factors with inconsistent reporting. Self-rated general health may serve as a proxy for multimorbidity and in line with our findings, lower general health status is associated with greater inconsistent reporting (Klabunde et al., 2005; Ryu et al., 2018). Our study builds on existing literature as we found multimorbidity is a primary predictor of longitudinal inconsistent self-reporting of chronic conditions.

Only one other known study by Cigolle and colleagues has adjudicated inconsistent self-reporting of responses in the 1995–2010 waves of the Health and Retirement study (*n* = 24,156) by using illness-related information (Cigolle et al., 2016). 30% of participants aged 51 years and older had inconsistently self-reported at least one of seven diseases across waves and they were able to adjudicate 60–75% of inconsistent responses (Cigolle et al., 2016). We used a similar adjudication method in our study (Method B) and were able to resolve >95% of inconsistent responses; the higher proportion corrected is likely because more illness-related information is collected in the CLSA which was used to adjudicate inconsistent responses. Directly asking participants to verify their inconsistent responses (Method A) was a more conservative method as up to 53% of responses were resolved. Future studies should seek to compare and validate these two methods of ascertaining disease status.

### Strengths and Limitations

Strengths of this study include the use of the large nationally representative CLSA dataset. Survey weights were applied to all analyses enabling our findings to be generalizable to the eligible Canadian population. We considered 35 chronic conditions which were commonly included in indices of multimorbidity and were strategically selected by collaborating with clinicians to determine conditions most relevant to aging (Fortin et al., 2012; Johnston et al., 2019). This number of conditions is within the recommended range for sufficient sensitivity to detect multimorbidity (Holzer et al., 2017). Additionally, participants self-reported chronic conditions in face-to-face interviews (Comprehensive cohort) and telephone interviews (Tracking cohort) which were conducted in a standardized manner by trained research staff. The use of computer-assisted survey software enabled a new adjudication method (Method A) to be explored and can inform the design of future longitudinal surveys.

Despite the strengths of our study, it is a limitation that illness-related information used to resolve inconsistent responses was not consistently available for all chronic conditions. The validity of using illness-related information to ascertain disease status (Method B) is unknown (Quiñones et al., 2020). A participant may mistakenly provide illness-related information, such as age at diagnosis, for a particular condition they do not have which may explain the high percentage of inconsistent responses that were resolved using Method B. Additionally, carrying a respondent’s baseline response forward to follow-up (Method C) may not be the most appropriate method for resolving responses as it has been demonstrated to lead to bias when estimating the effect of treatments on health outcomes (Molnar et al., 2008). The management of some conditions, such as type II diabetes, can result in remission, and other relapsing-remitting conditions vary with time and treatment. In these instances, a participant may report not having the condition if they misunderstand the specific wording of the question that asks if a doctor has *“ever”* told them they had a particular condition. Therefore, findings using these adjudication methods are exploratory and should be interpreted as such. Our study was only able to consider identifiable inconsistent responses between baseline and follow-up surveys. Other types of inconsistencies are possible (e.g., incorrectly reporting negative at baseline and affirmative at follow-up) but could not be identified in this study. It is also possible that an inconsistent response may be a result of a misdiagnosis which the respondent believed was present at baseline but understood not to be present at follow-up (Singh et al., 2014). Alternatively, the chronic condition may have been less symptomatic or better managed at follow-up—for instance, less low back pain or fewer symptoms of arthritis. Future research should seek to better understand the contributing causes of inconsistent responses which may reflect their understanding of their condition status at the time of interview, the quality of their care and/or health literacy.

## Conclusion

The current study found it was common for mid- to older-aged Canadian adults to inconsistently self-report chronic conditions between a baseline and follow-up survey ∼three years later, although these inconsistencies do not appear to substantially affect the prevalence of multimorbidity. Future studies should explore the validity of using illness-related information to ascertain disease status, especially among older adult populations and within studies on aging where inconsistent reporting of chronic conditions is common. The use of self-reported survey data represents one commonly used method of tracking chronic conditions over time; integration of diverse data sources, including medication or prescription data, laboratory values and diagnostic tests, as well as administrative health care data, can facilitate consistent tracking of chronic conditions over time and assist in the development of valid and reliable disease ascertainment algorithms.

## Supplemental Material

Supplemental Material - An Exploration of Methods to Resolve Inconsistent Self-Reporting of Chronic Conditions and Impact on Multimorbidity in the Canadian Longitudinal Study on Aging Supplemental Material for An Exploration of Methods to Resolve Inconsistent Self-Reporting of Chronic Conditions and Impact on Multimorbidity in the Canadian Longitudinal Study on Aging by Alessandra T. Andreacchi, Alberto Brini, Edwin Van Den Heuvel, Graciela Muniz-Terrera, Alexandra Mayhew, Philip St. John, Lucy E. Stirland, and Lauren E. Griffith in Journal of Aging and Health

## Data Availability

Data are available from the Canadian Longitudinal Study on Aging (www.clsa-elcv.ca) for researchers who meet the criteria for access to de-identified CLSA data.
