# Supplementary material for: An Exploration of Methods to Resolve Inconsistent Self-Reporting of Chronic Conditions and Impact on Multimorbidity in the Canadian Longitudinal Study on Aging
Source: J Aging Health. 2023 Nov 28;37(1-2):40–53. doi: 10.1177/08982643231215476 (PMC11566091; doi:10.1177/08982643231215476)
Supplement: Supplemental Material - An Exploration of Methods to Resolve Inconsistent Self-Reporting of Chronic Conditions and Impact on Multimorbidity in the Canadian Longitudinal Study on Aging [file sj-pdf-1-jah-10.1177_08982643231215476.pdf]

Supplemental Materials

eFigure 1. Visual schematic of the two identifiable types of inconsistent self-reported chronic conditions between baseline (2011-2015) and the first follow-up (2015-2018).

eFigure 2. Visual schematic of Method A to resolve inconsistent self-reported responses for participants responding affirmative at baseline (2011-2015) and then negative at follow-up (2015-2018) for a given chronic condition.

eFigure 3. Visual schematic of Method B to resolve inconsistent self-reported responses for participants responding affirmative at baseline (2011-2015) and then negative or unknown at follow-up (2015-2018) for a given chronic condition.

eTable 1. Information available at baseline for each chronic condition used in Methods A and B to resolve inconsistent responses. Illness-related information used in Method B included age at diagnosis, whether a participant was currently or had ever taken medication or undergone non-pharmacological treatment to treat the condition.

eTable 2. Baseline socio-demographic and health-related factors of participants in the Canadian Longitudinal Study on Aging Tracking cohort (n=17,429) who had no and at least one affirmative at baseline and negative at follow-up inconsistent response across all 35 chronic conditions.

eTable 3. Baseline cognitive impairment test scores across participants in the Canadian Longitudinal Study on Aging Comprehensive cohort (n=27,765) who had no and at least one affirmative at baseline and negative at follow-up inconsistent response across all 35 chronic conditions.

eTable 4. Baseline cognitive impairment test scores across participants in the Canadian Longitudinal Study on Aging Tracking cohort (n=17,429) who had no and at least one affirmative at baseline and negative at follow-up inconsistent response across all 35 chronic conditions.

eTable 5. Weighted odds ratios and 95% confidence intervals for the association between socio-demographic and health-related factors, and odds of at least one affirmative at baseline and then negative at follow-up inconsistent response (reference: no inconsistent responses) across all 35 chronic conditions among participants in the Canadian Longitudinal Study on Aging Tracking cohort (n=17,429).

eTable 6. Weighted odds ratios and 95% confidence intervals for the association between cognitive impairment test scores and the odds of at least one affirmative at baseline and then negative or unknown at follow-up inconsistent response (reference: no inconsistent responses) across all 35 chronic conditions among participants in the Canadian Longitudinal Study on Aging Comprehensive (n=27,765) and Tracking cohorts (n=17,429).

eTable 7. Frequency and proportion (%) of inconsistent responses resolved by Method A and Method B, separately, for each chronic condition in the Canadian Longitudinal Study on Aging Comprehensive cohort (n=27,765).

eFigure 1. Visual schematic of the two identifiable types of inconsistent self-reported chronic conditions between baseline (2011-2015) and the first follow-up (2015-2018).

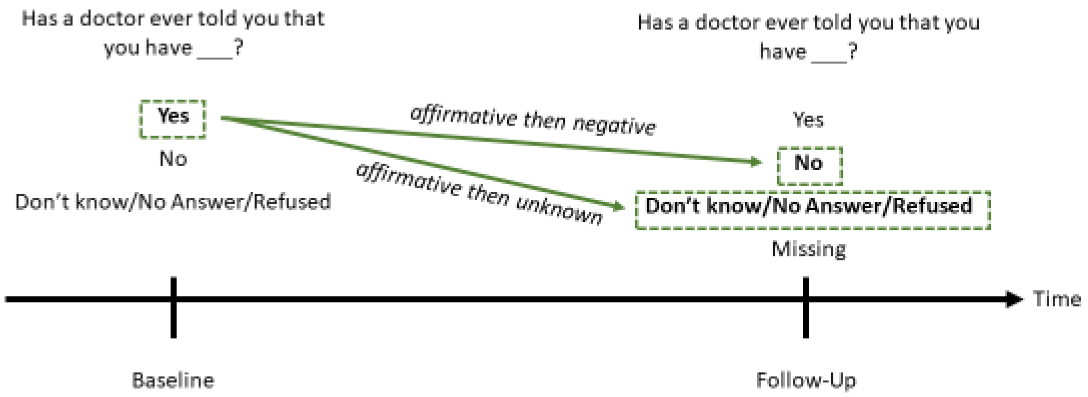

eFigure 2. Visual schematic of Method A to resolve inconsistent self-reported responses for participants responding affirmative at baseline (2011-2015) and then negative at follow-up (2015-2018) for a given chronic condition.

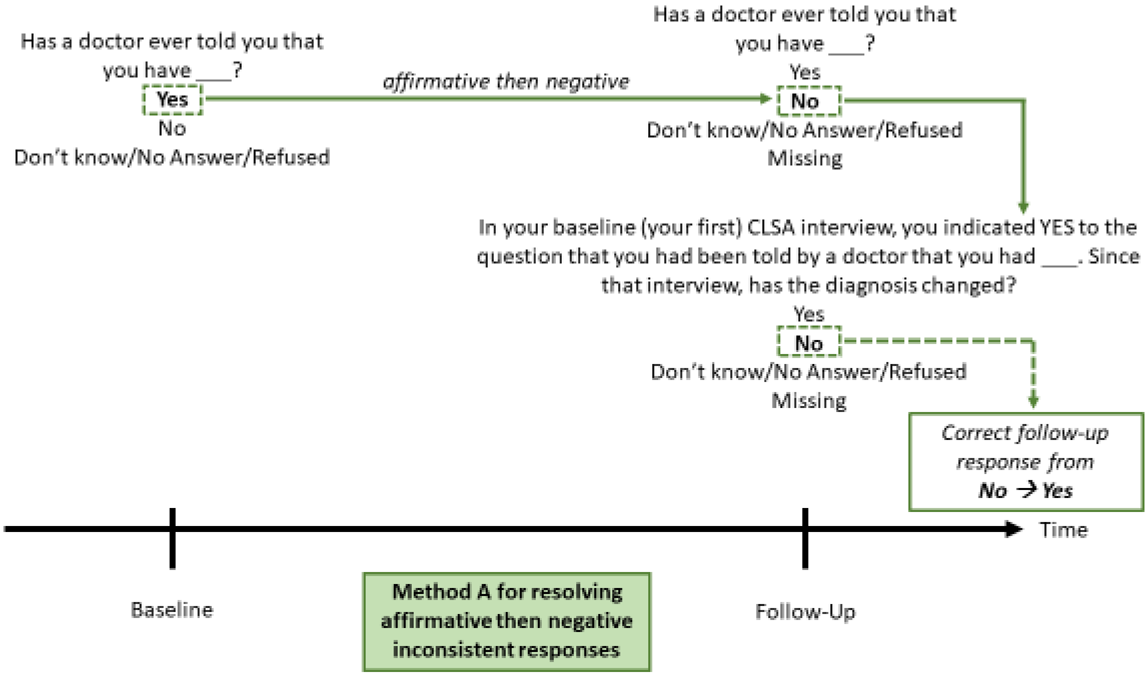

eFigure 3. Visual schematic of Method B to resolve inconsistent self-reported responses for participants responding affirmative at baseline (2011-2015) and then negative or unknown at follow-up (2015-2018) for a given chronic condition.

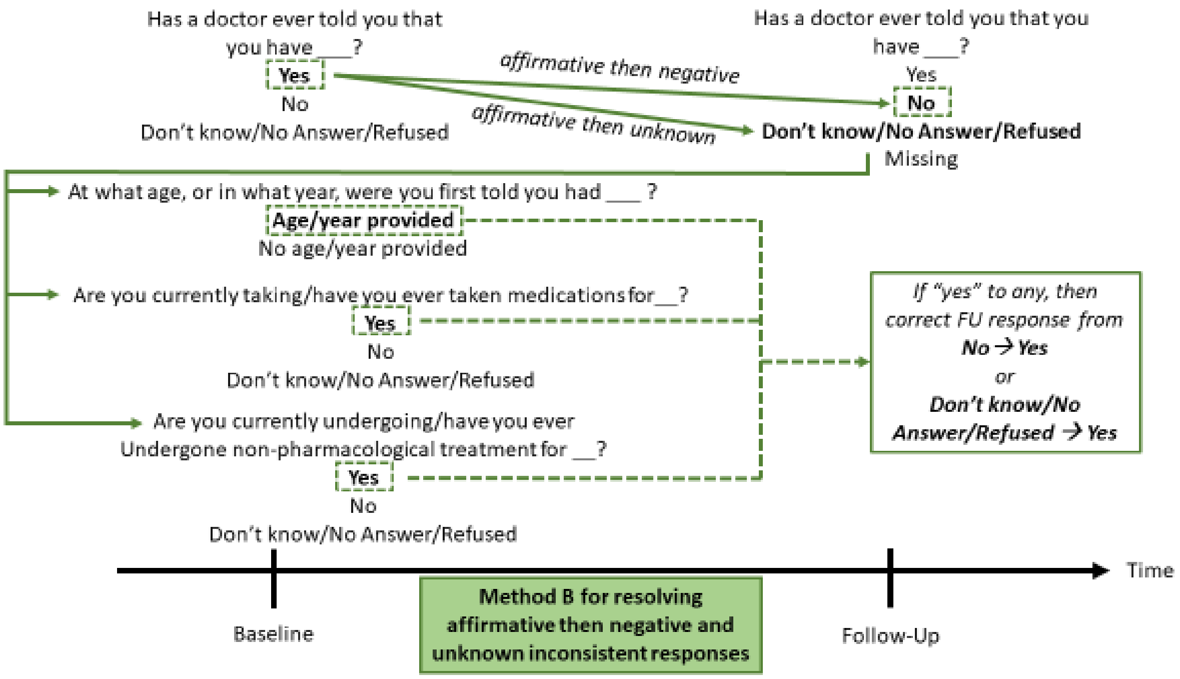

eTable 1. Information available at baseline for each chronic condition used in Methods A and B to resolve inconsistent responses. Illness-related information used in Method B included age at diagnosis, whether a participant was currently or had ever taken medication or undergone non-pharmacological treatment to treat the condition.

**CLSA Chronic Condition**	**Method A: Change in Diagnosis**	**Method B: Age of diagnosis, current/ever medication use, current/ever had non-pharmacological treatment for the condition**
Angina	X	Age
Anxiety	X	N/C
Asthma	X	Age
Back Problems	X	N/C
Bowel disorder	X	N/C
Bowel incontinence	N/C	N/C
Cancer (excluding non-melanoma)	X	N/C
Cataracts	N/C	N/C
Chronic obstructive pulmonary disorder	X	Age
Clinical depression	X	Age, current medication, ever medication/non-pharmacological treatment, current non-pharmacological treatment
Diabetes	X	Age, current medication
Dementia including Alzheimer's disease	X	N/C
Epilepsy	X	N/C
Glaucoma	N/C	N/C
Heart disease (including CHF)	X	N/C
Hypertension	X	Age, current medication, ever medication, current non-pharmacological treatment, ever non- pharmacological treatment
Hyperthyroidism	X	Age, current medication, ever medication
Hypothyroidism	X	Age, current medication, ever medication
Intestinal or stomach ulcer	X	N/C
Kidney disease	X	N/C
Macular Degeneration	X	N/C

CLSA Chronic Condition	Method A: Change in Diagnosis	Method B: Age of diagnosis, current/ever medication use, current/ever had non-pharmacological treatment for the condition
Migraine headaches	X	N/C
Mood disorder (depression, bipolar, mania, dysthymia)	X	N/C
Myocardial infarction	X	Age
Multiple Sclerosis	X	N/C
Osteoarthritis in hand	X	Age
Osteoarthritis in hip	X	Age
Osteoarthritis in knee	X	Age
Osteoporosis	X	Age, current medication, ever medication
Parkinsonism/disease	X	Age, current medication, ever medication/non-pharmacological, current non-pharmacological treatment
Peripheral vascular disease	X	N/C
Rheumatoid arthritis	X	N/C
Stroke	X	Age, current medication, ever medication/non-pharmacological treatment, current non-pharmacological treatment
Transient ischemic attack	X	Age, current medication, ever medication/non-pharmacological treatment, current non-pharmacological treatment
Urinary incontinence	N/C	N/C

- Note: N/C = not collected.

eTable 2. Baseline socio-demographic and health-related factors of participants in the Canadian Longitudinal Study on Aging Tracking cohort (n=17,429) who had no and at least one affirmative at baseline and negative at follow-up inconsistent response across all 35 chronic conditions.

**Socio-demographic and health-related factors**	**No Inconsistent Responses** N=10,523 (60.4%)		**≥1 Inconsistent Response** N=6,906 (39.6%)		**Chi-square p-value**	**SD**	**Overall** N=17,429	
	**N**	**%**	**N**	**%**			**N**	**%**
Sex								
Male	5,291	50.3	3,141	45.5	<0.0001	0.10	8,432	48.4
Female	5,232	49.7	3,765	54.5		0.10	8,997	51.6
Missing	0	0.0	0	0.0		0.00	0	0.0
Age Group								
45-54	3,489	33.2	1,431	20.7	<0.0001	0.28	4,920	28.2
55-64	3,526	33.5	2,053	29.7		0.08	5,579	32.0
65-74	2,080	19.8	1,769	25.6		0.14	3,849	22.1
75+	1,419	13.5	1,653	23.9		0.27	3,072	17.6
Missing	9	0.1	0	0.0		0.01	9	0.1
Race/Ethnicity								
White	10,209	97.0	6,714	97.2	0.5923	0.01	16,923	97.1
Not White	303	2.9	183	2.6		0.01	486	2.8
Missing	11	0.1	9	0.1		0.01	20	0.1
Immigrant Status								
Non-immigrant	9,208	87.5	5,970	86.4	0.0849	0.03	15,178	87.1
Immigrant	1,314	12.5	934	13.5		0.03	2,248	12.9
Missing	1	0.0	2	0.0		0.01	3	0.0
Province								
Alberta	1,105	10.5	671	9.7	<0.0001	0.03	1,776	10.2
British Columbia	1,393	13.2	843	12.2		0.03	2,236	12.8
Manitoba	731	6.9	462	6.7		0.01	1,193	6.8
New Brunswick	654	6.2	434	0.1		0.00	1,088	6.2
Newfoundland and Labrador	557	5.3	388	5.6		0.01	945	5.4
Nova Scotia	731	6.9	537	7.8		0.03	1,268	7.3
Ontario	2,272	21.6	1,623	23.5		0.05	3,895	22.3
Prince Edward Island	511	4.9	396	5.7		0.04	907	5.2
Quebec	1,917	18.2	1,087	15.7		0.07	3,004	17.2
Saskatchewan	643	6.1	465	6.7		0.03	1,108	6.4
Missing	9	0.1	0	0.0		0.01	9	0.1
Highest level of education								
Less than secondary school	689	6.5	646	9.4	<0.0001	0.10	1,335	7.7

**Socio-demographic and health-related factors**	**No Inconsistent Responses** N=10,523 (60.4%)		**≥1 Inconsistent Response** N=6,906 (39.6%)		**Chi-square p-value**	**SD**	**Overall** N=17,429	
	**N**	**%**	**N**	**%**			**N**	**%**
Secondary school graduation	1,351	12.8	907	13.1		0.01	2,258	13.0
Some post-secondary education	755	7.2	564	8.2		0.04	1,319	7.6
Post-secondary degree/diploma	7,685	73.0	4,760	68.9		0.09	12,445	71.4
Missing	43	0.4	29	0.4		0.00	72	0.4
Household income								
Less than $20,000	445	4.2	469	6.8	<0.0001	0.11	914	5.2
$20,000 or more, but less than $50,000	2,446	23.2	2,066	29.9		0.15	4,512	25.9
$50,000 or more, but less than $100,000	3,718	35.3	2,377	34.4		0.02	6,095	35.0
$100,000 or more, but less than $150,000	1,874	17.8	953	13.8		0.11	2,827	16.2
$150,000 or more	1,428	13.6	580	8.4		0.17	2,008	11.5
Missing	612	5.8	461	6.7		0.04	1,073	6.2
Marital status								
Single/Never married	814	7.7	537	7.8	<0.0001	0.00	1,351	7.8
Married/Common law/	7,677	73.0	4,587	66.4		0.14	12,264	70.4
Widowed	836	7.9	887	12.8		0.16	1,723	9.9
Divorced/Separated	1,191	11.3	894	12.9		0.05	2,085	12.0
Missing	5	0.0	1	0.0		0.02	6	0.0
Interview language								
English	8,528	81.0	5,751	83.3	0.0002	0.06	14,279	81.9
French	1,995	19.0	1,155	16.7		0.06	3,150	18.1
Missing	0	0.0	0	0.0		0.00	0	0.0
Contact with general practitioner in past 12 months								
No	1,191	11.3	484	7.0	<0.0001	0.15	1,675	9.6
Yes	9,117	86.6	6,312	91.4		0.15	15,429	88.5
Missing	215	2.0	110	1.6		0.03	325	1.9
Self-reported general health								
Fair/Poor	854	8.1	1,140	16.5	<0.0001	0.20	1,994	11.4
Good	2,864	27.2	2,220	32.1		0.11	5,084	29.2
Very good	4,308	40.9	2,570	37.2		0.08	6,878	39.5
Excellent	2,488	23.6	971	14.1		0.25	3,459	19.8
Missing	9	0.1	5	0.1		0.00	14	0.1

- Note: SD = standardized difference.

eTable 3. Baseline cognitive impairment test scores across participants in the Canadian Longitudinal Study on Aging Comprehensive cohort (n=27,765) who had no and at least one affirmative at baseline and negative at follow-up inconsistent response across all 35 chronic conditions.

**Cognitive Impairment Tests**	**No Inconsistent Responses** N=18,858 (67.9%)		**≥1 Inconsistent Responses** N=8,907 (32.1%)		**Chi-square p-value**	**SD**	**Overall** N=27,765	
	**N**	**%**	**N**	**%**			**N**	**%**
**First Recall RAVLT T-score**					<0.0001			
Moderate to severe impairment	824	4.4	468	5.3		0.01	1,292	4.7
Moderate impairment	4,294	22.8	2,044	22.9		0.03	6,338	22.8
Mild impairment	7,276	38.6	3,495	39.2		0.02	10,771	38.8
Below average or borderline impairment	4,369	23.2	1,900	21.3		0.01	6,269	22.6
Average	1,480	7.8	663	7.4		0.03	2,143	7.7
Missing	615	3.3	337	3.8		0.04	952	3.4
**Second Recall RAVLT T-score**					0.0007			
Moderate to severe impairment	987	5.2	489	5.5		0.01	1,476	5.3
Moderate impairment	3,969	21.0	1,981	22.2		0.03	5,950	21.4
Mild impairment	7,485	39.7	3,468	38.9		0.02	10,953	39.4
Below average or borderline impairment	4,415	23.4	2,039	22.9		0.01	6,454	23.2
Average	1,397	7.4	582	6.5		0.03	1,979	7.1
Missing	605	3.2	348	3.9		0.04	953	3.4
**Animal Naming T-score**					0.0005			
Moderate to severe impairment	930	4.9	448	5.0		0.00	1,378	5.0
Moderate impairment	4,180	22.2	2,135	24.0		0.04	6,315	22.7
Mild impairment	7,242	38.4	3,433	38.5		0.00	10,675	38.4
Below average or borderline impairment	4,545	24.1	2,014	22.6		0.04	6,559	23.6
Average	1,516	8.0	641	7.2		0.03	2,157	7.8
Missing	445	2.4	236	2.6		0.02	681	2.5
**MAT T-score**					0.0124			
Moderate to severe impairment	955	5.1	458	5.1		0.00	1,413	5.1
Moderate impairment	3,535	18.7	1,760	19.8		0.03	5,295	19.1
Mild impairment	7,363	39.0	3,449	38.7		0.01	10,812	38.9
Below average or borderline impairment	5,015	26.6	2,287	25.7		0.02	7,302	26.3
Average	1,113	5.9	477	5.4		0.02	1,590	5.7
Missing	877	4.7	476	5.3		0.03	1,353	4.9

- Note: SD = standardized difference; RAVLT = Rey Auditory Verbal Learning Test; MAT = Mental Alternation Test.

eTable 4. Baseline cognitive impairment test scores across participants in the Canadian Longitudinal Study on Aging Tracking cohort (n=17,429) who had no and at least one affirmative at baseline and negative at follow-up inconsistent response across all 35 chronic conditions.

**Cognitive Impairment Tests**	**No Inconsistent Responses** N=10,523 (60.4%)		**≥1 Inconsistent Response** N=6,906 (39.6%)		**Chi-square p-value**	**SD**	**Overall** N=17,429	
	**N**	**%**	**N**	**%**			**N**	**%**
**First Recall RAVLT T-score**					<0.0001			
Moderate to severe impairment	280	2.7	221	3.2		0.04	501	2.9
Moderate impairment	2,394	22.8	1,691	24.5		0.06	4,085	23.4
Mild impairment	4,180	39.7	2,670	38.7		0.01	6,850	39.3
Below average or borderline impairment	2,031	19.3	1,199	17.4		0.09	3,230	18.5
Average	862	8.2	488	7.1		0.02	1,350	7.7
Missing	776	7.4	637	9.2		0.04	1,413	8.1
**Second Recall RAVLT T-score**					<0.0001			
Moderate to severe impairment	306	2.9	225	3.3		0.04	531	3.1
Moderate impairment	2,299	21.8	1,676	24.4		0.06	3,975	22.8
Mild impairment	4,185	39.8	2,708	39.4		0.01	6,893	39.6
Below average or borderline impairment	2,057	19.5	1,119	16.3		0.09	3,176	18.3
Average	839	8.0	519	7.5		0.02	1,358	7.8
Missing	837	8.0	629	9.1		0.04	1,466	8.4
**Animal Naming T-score**					<0.0001			
Moderate to severe impairment	481	4.6	320	4.6		0.00	801	4.6
Moderate impairment	2,272	21.6	1,577	22.8		0.03	3,849	22.1
Mild impairment	3,871	36.8	2,586	37.4		0.01	6,457	37.0
Below average or borderline impairment	2,657	25.2	1,588	23.0		0.05	4,245	24.4
Average	874	8.3	509	7.4		0.03	1,383	7.9
Missing	368	3.5	326	4.7		0.06	694	4.0
**MAT T-score**					<0.0001			
Moderate to severe impairment	501	4.9	375	5.4		0.03	876	5.1
Moderate impairment	1,551	15.1	1,126	16.3		0.04	2,677	15.6
Mild impairment	3,965	38.7	2,521	36.5		0.02	6,486	37.8
Below average or borderline impairment	2,097	20.5	1,743	25.2		0.05	3,840	22.4
Average	502	4.9	285	4.1		0.03	787	4.6
Missing	1,631	15.9	856	12.4		0.06	2,487	14.5

- Note: SD = standardized difference; RAVLT = Rey Auditory Verbal Learning Test; MAT = Mental Alternation Test.

eTable 5. Weighted odds ratios and 95% confidence intervals for the association between socio-demographic and health-related factors, and odds of at least one affirmative at baseline and then negative at follow-up inconsistent response (reference: no inconsistent responses) across all 35 chronic conditions among participants in the Canadian Longitudinal Study on Aging Tracking cohort (n=17,429).

Socio-demographic, illness-related, and cognitive factors	Sample size	Unadjusted			Adjusted for sex and age (continuous)		
		OR	95% CI		OR	95 % CI	
Sex	17,429						
Male		Ref			Ref		
Female		1.23	1.15	1.32	1.21	1.13	1.30
Age group	17,429						
45-54		Ref			Ref		
55-64		1.44	1.31	1.58	1.44	1.31	1.58
65-74		2.11	1.91	2.32	2.10	1.91	2.32
75+		2.97	2.67	3.30	2.94	2.65	3.27
Race	17,429						
White		Ref			Ref		
Not White		0.88	0.72	1.08	0.99	0.80	1.22
Immigrant status	17,426						
Non-immigrant		Ref			Ref		
Immigrant		1.00	0.90	1.11	0.94	0.85	1.05
Province	17,420						
Alberta		0.87	0.75	1.00	0.90	0.78	1.04
British Columbia		0.80	0.71	0.91	0.79	0.69	0.90
Manitoba		0.93	0.80	1.08	0.91	0.78	1.06
New Brunswick		0.93	0.81	1.08	0.93	0.80	1.08
Newfoundland and Labrador		1.00	0.86	1.17	1.03	0.88	1.21
Nova Scotia		1.08	0.94	1.24	1.08	0.94	1.25
Ontario		Ref			Ref		
Prince Edward Island		1.07	0.92	1.25	1.06	0.90	1.24
Quebec		0.83	0.74	0.93	0.82	0.73	0.92
Saskatchewan		1.02	0.89	1.18	1.00	0.87	1.16
Education	17,357						
Less than secondary school graduation		1.65	1.45	1.87	1.29	1.13	1.47
Secondary school graduation, no post-secondary education		1.14	1.03	1.26	1.08	0.97	1.19
Some post-secondary education		1.25	1.09	1.42	1.18	1.03	1.35
Post-secondary degree/diploma		Ref			Ref		
Household income	16,356						
Less than $20,000		2.74	2.28	3.29	1.99	1.64	2.41
$20,000 or more, but less than $50,000		2.21	1.94	2.51	1.52	1.32	1.74
$50,000 or more, but less than $100,000		1.64	1.44	1.86	1.33	1.17	1.51
$100,000 or more, but less than $150,000		1.30	1.12	1.49	1.22	1.06	1.41
$150,000 or more		Ref			Ref		
Marital status	17,423						
Single/Never married		1.19	1.04	1.36	1.25	1.09	1.44
Married/Common law		Ref			Ref		
Widowed		1.85	1.66	2.07	1.10	0.98	1.25

Socio-demographic, illness-related, and cognitive factors	Sample size	Unadjusted			Adjusted for sex and age (continuous)		
		OR	95% CI		OR	95 % CI	
Divorced/Separated		1.27	1.14	1.41	1.18	1.06	1.32
Interview language	17,429						
French		0.89	0.81	0.98	0.90	0.82	0.99
English		Ref			Ref		
Visit to general practitioner in past 12 months	17,104						
No		Ref			Ref		
Yes		1.60	1.41	1.82	1.40	1.23	1.59
Self-reported general health	17,415						
Fair/Poor		3.63	3.18	4.14	3.65	3.19	4.18
Good		1.97	1.77	2.19	1.96	1.76	2.18
Very good		1.53	1.38	1.69	1.53	1.38	1.70
Excellent		Ref			Ref		
Number of chronic conditions	17,429	1.63	1.60	1.67	1.63	1.60	1.67

- Note: OR = odds ratio; CI = confidence interval; ref = reference.

eTable 6. Weighted odds ratios and 95% confidence intervals for the association between cognitive impairment test scores and the odds of at least one affirmative at baseline and then negative or unknown at follow-up inconsistent response (reference: no inconsistent responses) across all 35 chronic conditions among participants in the Canadian Longitudinal Study on Aging Comprehensive (n=27,765) and Tracking cohorts (n=17,429).

Cognitive impairment tests	Sample size	OR[a]	95% CI	
**Comprehensive cohort**				
First Recall RAVLT T-score	26,813			
Moderate to Severe Impairment		1.22	1.03	1.44
Moderate Impairment		1.04	0.92	1.17
Mild Impairment		1.08	0.97	1.21
Below Average or Borderline Impairment		0.98	0.87	1.11
Average		Ref		
Second Recall RAVLT T-score	26,812			
Moderate to Severe Impairment		1.19	1.01	1.40
Moderate Impairment		1.12	0.99	1.27
Mild Impairment		1.07	0.95	1.20
Below Average or Borderline Impairment		1.09	0.97	1.24
Average		Ref		
Animal Naming T-score	27,084			
Moderate to Severe Impairment		1.13	0.96	1.34
Moderate Impairment		1.22	1.09	1.38
Mild Impairment		1.13	1.01	1.27
Below Average or Borderline Impairment		1.06	0.94	1.19
Average		Ref		
MAT T-score	26,412			
Moderate to Severe Impairment		1.02	0.85	1.22
Moderate Impairment		1.11	0.97	1.28
Mild Impairment		1.03	0.90	1.17
Below Average or Borderline Impairment		1.04	0.91	1.19
Average		Ref		
**Tracking cohort**				
First Recall RAVLT T-score	16,016			
Moderate to Severe Impairment		1.47	1.16	1.85
Moderate Impairment		1.24	1.08	1.43
Mild Impairment		1.09	0.96	1.25
Below Average or Borderline Impairment		1.03	0.89	1.20
Average		Ref		
Second Recall RAVLT T-score	15,963			
Moderate to Severe Impairment		1.35	1.08	1.68
Moderate Impairment		1.16	1.00	1.33
Mild Impairment		1.02	0.89	1.16
Below Average or Borderline Impairment		0.87	0.75	1.01
Average		Ref		
Animal Naming T-score	16,735			
Moderate to Severe Impairment		1.07	0.87	1.30

**Cognitive impairment tests**	**Sample size**	**OR**[a]	**95% CI**	
Moderate Impairment		1.13	0.98	1.30
Mild Impairment		1.10	0.96	1.26
Below Average or Borderline Impairment		0.99	0.86	1.14
Average		Ref		
MAT T-score	15,476			
Moderate to Severe Impairment		1.32	1.05	1.67
Moderate Impairment		1.29	1.07	1.57
Mild Impairment		1.13	0.95	1.36
Below Average or Borderline Impairment		1.05	0.87	1.26
Average		Ref		

- Note: OR = odds ratio; CI = confidence interval; ref = reference; RAVLT = Rey Auditory Verbal Learning Test; MAT = Mental Alternation Test.

[a] Odds ratios are unadjusted as cognitive impairment test T-scores are sex- and age-standardized.

eTable 7. Frequency and proportion (%) of inconsistent responses resolved by Method A and Method B, separately, for each chronic condition in the Canadian Longitudinal Study on Aging Comprehensive cohort (n=27,765).

	Affirmative at baseline, negative at follow-up	Affirmative at baseline, unknown at follow-up	Resolved affirmative then negative inconsistent responses by Method A		Resolved affirmative then negative inconsistent responses by Method B		Resolved affirmative then unknown inconsistent responses by Method B	
**Chronic Conditions**	**N**	**N**	**N**	**%**	**N**	**%**	**N**	**%**
Angina	189	25	40	21.2	185	97.9	25	100.0
Anxiety	393	26	88	22.4	N/C		N/C	
Asthma	297	45	48	16.2	290	97.6	45	100.0
Back problems	1174	65	232	19.8	N/C		N/C	
Bowel disorder	457	35	86	18.8	N/C		N/C	
Bowel incontinence	188	1	N/C		N/C		N/C	
Cancer (excluding non-melanoma)	221	15	17	7.7	N/C		N/C	
Cataracts	839	39	N/C		N/C		N/C	
Chronic obstructive pulmonary disorder	310	32	68	21.9	299	96.5	31	96.9
Clinical depression	958	112	158	16.5	956	99.8	112	100.0
Diabetes	416	20	19	4.6	414	99.5	26	100.0
Dementia including Alzheimer's disease	19	2	5	26.3	N/C		N/C	
Epilepsy	34	0	3	8.8	N/C		N/C	
Glaucoma	342	21	N/C		N/C		N/C	
Heart disease	472	32	100	21.2	N/C		N/C	
Hypertension	622	60	107	17.2	618	99.4	60	100.0
Hyperthyroidism	128	26	26	20.3	119	93.0	61	100.0
Hypothyroidism	176	61	94	53.4	167	94.9	20	100.0
Intestinal or stomach ulcer	419	26	72	17.2	N/C		N/C	
Kidney disease	182	8	34	18.7	N/C		N/C	
Macular Degeneration	181	30	30	16.6	N/C		N/C	
Migraine headaches	458	20	72	15.7	N/C		N/C	
Mood disorder (depression, bipolar, mania, dysthymia)	562	30	112	19.9	N/C		N/C	
Myocardial infarction	86	28	6	7.0	80	93.0	28	100.0
Multiple Sclerosis	19	1	5	26.3	N/C		N/C	
Osteoarthritis in hand	602	111	117	19.4	582	96.7	104	93.7
Osteoarthritis in hip	425	103	78	18.4	415	97.6	100	97.1
Osteoarthritis in knee	481	132	89	18.5	475	98.8	130	98.5
Osteoporosis	401	68	62	15.5	52	13.0	0	0.0
Parkinsonism/disease	5	0	0	0.0	5	100.0	0	0.0
Peripheral vascular disease	367	20	90	24.5	N/C		N/C	
Rheumatoid arthritis	154	39	22	14.3	N/C		N/C	
Stroke or CVA	87	12	7	8.0	86	98.9	12	100.0
Transient ischemic attack	125	34	23	18.4	123	98.4	32	94.1
Urinary incontinence	588	4	N/C		N/C		N/C	

- Note: N/C= not collected.
